# A Biopolymeric Dextran-Chitosan Delivery System for Controlled Release of Antioxidant and Anti-Inflammatory Compounds: Lignin and Curcumin

**DOI:** 10.3390/molecules30061276

**Published:** 2025-03-12

**Authors:** Paula Cucu, Violeta Melinte, Anca Roxana Petrovici, Narcis Anghel, Irina Apostol, Mihai Mares, Natalia Simionescu, Iuliana Spiridon

**Affiliations:** 1 Faculty of Veterinary Medicine, “Ion Ionescu de la Brad” University of Life Sciences, 700490 Iasi, Romania; paula.cucu@iuls.ro (P.C.); mihai.mares@iuls.ro (M.M.); 2 “Petru Poni” Institute of Macromolecular Chemistry, 700487 Iasi, Romaniapetrovici.anca@icmpp.ro (A.R.P.); apostol.irina@icmpp.ro (I.A.); natalia.simionescu@icmpp.ro (N.S.)

**Keywords:** chitosan, dextran, curcumin, medical applications

## Abstract

Biopolymeric drug delivery systems enhance the bioavailability and therapeutic efficacy of poorly soluble bioactive compounds. In this study, chitosan (Chi), dextran (Dex), carboxymethyl dextran (mDex), lignin (L), and curcumin (Cu) were combined to develop materials with controlled release, antioxidant, and anti-inflammatory properties. The mechanical evaluation showed that Chi-mDex-L-Cu exhibited the highest diametral tensile strength (2.40 MPa), a 1233% increase compared to Chi-mDex-L, due to strong hydrogen bonding interactions between curcumin and matrix components. Curcumin release kinetics, modeled using the Weibull equation, demonstrated that Chi-mDex-L-Cu presented the slowest release rate, reducing the cumulative release by 55.66% as compared to Chi-L-Cu, ensuring prolonged bioactivity. Despite its controlled release, Chi-mDex-L-Cu retained 60% antioxidant and 70% anti-inflammatory activity, making it a promising sustained-release system. The biocompatibility assessment confirmed cell viability above 85%, with Chi-mDex-L-Cu showing a slight (~10%) reduction at higher concentrations while remaining non-cytotoxic. These findings suggest that Chi-mDex-L-Cu is a strong candidate for biomedical applications requiring prolonged therapeutic effects, such as osteoarthritis treatment.

## 1. Introduction

The development of biopolymeric delivery systems has gained significant attention in pharmaceutical and biomedical research due to their biocompatibility, biodegradability, and versatility in drug encapsulation and release [1]. Natural polysaccharides represent a renewable and abundant class of biopolymers that have been widely explored for various medical and cosmetic applications [2]. Their inherent properties, including biocompatibility and ease of functionalization, make them suitable candidates for designing novel drug delivery platforms. Among these, chitosan and dextran have emerged as promising materials due to their unique physicochemical and biological properties [3,4].

Chitosan (Chi), a deacetylated derivative of chitin, is extensively studied for its antibacterial and antifungal activities, biocompatibility, and non-toxicity [5,6]. It has been employed in various drug delivery systems, including nanoparticles, microparticles, and hydrogels, to enhance drug solubility, stability, and bioavailability [7,8,9]. Additionally, chitosan is known to facilitate wound healing by promoting cell proliferation, migration, and differentiation [10,11]. Its ability to degrade in the human body via enzymatic hydrolysis, coupled with hemostatic, antimicrobial, and osteoconductive properties [12], makes it a valuable material in tissue engineering for regenerating bone, cartilage, and skin tissues [13,14,15].

Dextran (Dex) is another polysaccharide with significant biomedical potential. Its hydrophilic nature and capacity to form complexes with other natural polymers make this polymer useful in diverse applications, including blood plasma expansion [16], anticoagulant therapy [17], and antiviral treatments. Moreover, dextran and its derivatives have been employed in drug delivery systems to improve the controlled release of therapeutic agents [18,19,20,21,22]. Dextran’s ability to be chemically modified allows it to function as an efficient drug carrier, improving drug loading and controlled release profiles. Furthermore, dextran-based nanoparticles have demonstrated enhanced targeting capabilities, contributing to the development of effective drug delivery mechanisms [23,24,25].

Lignin (L), a natural aromatic macromolecule, provides structural support to plants and possesses remarkable antioxidant properties due to its phenolic pharmacophore, which enables it to scavenge reactive radical species [26,27,28,29]. This property allows lignin to create stable mesomeric forms, making it a valuable component in antioxidant formulations. The integration of lignin into polymeric drug delivery systems enhances their structural stability and improves their ability to neutralize oxidative stress, which is a major contributor to various chronic diseases and aging-related conditions.

Curcumin (Cu), a polyphenol derived from *Curcuma longa*, is well known for its antioxidant and anti-inflammatory activities [30,31,32]. It has been reported to target various biomolecules, including growth factors, cytokines, transcription factors, and enzymes, influencing cellular processes such as apoptosis and proliferation [33,34]. Despite its therapeutic potential, curcumin’s clinical application is hindered by its poor solubility in water, limited absorption, and short biological half-life. Strategies such as encapsulation in polymeric matrices have been explored to overcome these limitations and improve its bioavailability. It was reported [35] that curcumin-loaded liposomes can suppress tumor growth, highlighting its potential in cancer therapy.

The combination of these biopolymers presents an opportunity to develop multifunctional delivery systems with enhanced properties. By leveraging the antimicrobial and wound-healing capabilities of chitosan, the drug-loading efficiency of dextran, the antioxidant potential of lignin, and the bioactivity of curcumin, a synergistic approach can be employed to develop advanced therapeutic formulations. Such an approach enables the creation of a drug delivery system that not only ensures sustained release but also provides additional health benefits by modulating oxidative stress and inflammation.

A major challenge in drug delivery research is achieving a balance between controlled release and maintaining bioactivity over time. Encapsulation of curcumin within a dextran-chitosan-lignin matrix addresses this issue by protecting curcumin from degradation while allowing its gradual release at the target site. This controlled release mechanism enhances therapeutic outcomes by ensuring prolonged exposure to the bioactive compound, thereby increasing its efficacy. Additionally, the biopolymeric system provides protection against premature degradation by external environmental factors such as pH fluctuation and enzymatic breakdown, improving drug stability and shelf-life.

Polysaccharide-based drug delivery systems also offer advantages in terms of targeting specific tissues. Chitosan, for instance, exhibits mucoadhesive properties, enabling it to interact with mucosal surfaces and improve drug retention at the site of application [36,37]. This property is particularly valuable in the development of localized treatments, such as wound dressings and oral drug delivery formulations. Dextran, on the other hand, can be modified to enhance targeting capabilities, allowing selective delivery of therapeutic agents to diseased tissues while minimizing systemic side effects.

The developed materials were characterized in terms of structural, mechanical and biological properties.

From a material characterization perspective, Fourier-Transform Infrared Spectroscopy (FTIR) and Scanning Electron Microscopy (SEM) are crucial techniques for evaluating the physicochemical properties of the developed delivery system. FTIR provides insights into the molecular interactions between chitosan, dextran, lignin, and curcumin, confirming the successful incorporation of bioactive components. SEM, on the other hand, allows visualization of the surface morphology and structural integrity of the obtained materials, aiding in the assessment of their suitability for biomedical applications.

Mechanical testing is another essential aspect of material characterization. An optimal drug delivery system must possess sufficient mechanical strength to withstand handling and physiological conditions while maintaining its structural integrity throughout the drug release process. Evaluating mechanical properties ensures that the developed formulation meets the necessary standards for biomedical applications.

The evaluation of biological activities, including antioxidant and anti-inflammatory effects, provides opportunities for future validation of the potential therapeutic benefits of the developed system. Oxidative stress plays a central role in the pathogenesis of numerous diseases, and by incorporating lignin and curcumin into the biopolymeric matrix, the system can help mitigate these effects [38,39]. Anti-inflammatory assays assess the ability of the materials to reduce inflammation markers, offering insights into its potential applications in treating inflammatory disorders and promoting tissue regeneration.

The proliferative effects of fillers were assessed using the MTT assay (a colorimetric method for assessing cell metabolic activity). This assay provides valuable information regarding the cytocompatibility of the developed systems and their potential influence on cell growth. Ensuring that the biopolymeric formulation supports cellular activities without inducing cytotoxicity is a crucial step in advancing its application in medical therapies.

Furthermore, the release kinetics of curcumin were investigated to determine the effectiveness of the biopolymeric system in achieving sustained drug delivery. Controlled release mechanisms play a pivotal role in optimizing therapeutic outcomes, preventing the need for frequent dosing, and reducing potential side effects associated with fluctuating drug levels in the body. Understanding the release profile of curcumin helps in fine-tuning the formulation for specific medical applications.

In summary, this study presents the development of a novel biopolymeric delivery system based on chitosan, dextran/modified dextran, lignin, and curcumin. Dextran was chemically modified in order to obtain carboxymethyl dextran, while chitosan was crosslinked with glutaraldehyde and subsequently combined with dextran or carboxymethyl dextran to develop polymeric materials for bioactive principle delivery systems. The drug release rate was balanced by carboxymethyl dextran through crosslinking via electrostatic interactions between carboxyl and amino groups from chitosan, resulting in a more compact structure that effectively retains and sustains drug release.

By leveraging the unique properties of these biopolymers, the system offers a promising approach for the controlled release of antioxidant and anti-inflammatory compounds. The comprehensive characterization of the materials highlights their potential biomedical applications. The findings of this research contribute to the growing field of biopolymeric drug delivery systems, paving the way for innovative therapeutic solutions in medicine.

## 2. Results and Discussion

### 2.1. Fourier Transform Infrared (FTIR) Spectroscopy

**Figure 1:**
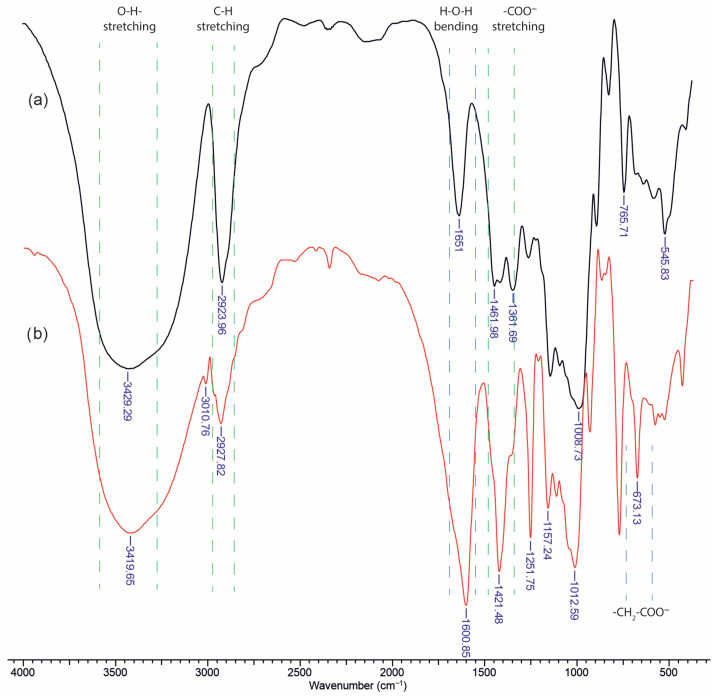
FTIR spectra of: (**a**) Dex; (**b**) mDex.

Infrared analysis of unsubstituted dextran (Figure 1a) reveals several characteristic polysaccharide bands, including a broad O–H stretching vibration in the region of 3400–3500 cm^−1^, which is indicative of extensive hydrogen bonding. Polysaccharides also tend to strongly retain water, leading to an H–O–H bending vibration commonly observed in the 1640–1660 cm^−1^ region. In the case of unmodified dextran, the band near 1650 cm^−1^ arises primarily from bound water rather than from functional groups of the polymer itself. Since there is no intrinsic carbonyl or amide group in dextran, any apparent absorption in that region is generally attributed to retained moisture.

Upon carboxymethylation of dextran (Figure 1b), new prominent features appear in both the high- and low-wavenumber regions. Notably, additional absorptions emerge near 1600 cm^−1^ and 1400 cm^−1^, corresponding to the asymmetric and symmetric stretching vibrations of the carboxylate group (–COO^−^), respectively. Furthermore, a distinct band becomes visible around 673 cm^−1^, which is attributed to an out-of-plane deformation of the glucopyranose ring. This band is only weakly observed or absent in unsubstituted dextran, but the introduction of –CH_2_–COO^−^ substituents modify the ring environment and hydrogen-bonding patterns, thereby enhancing or “activating” this low-frequency mode. Overall, the appearance of these new vibrational signals confirms successful carboxymethylation and provides insights into the altered molecular interactions within the polysaccharide chain.

**Figure 2:**
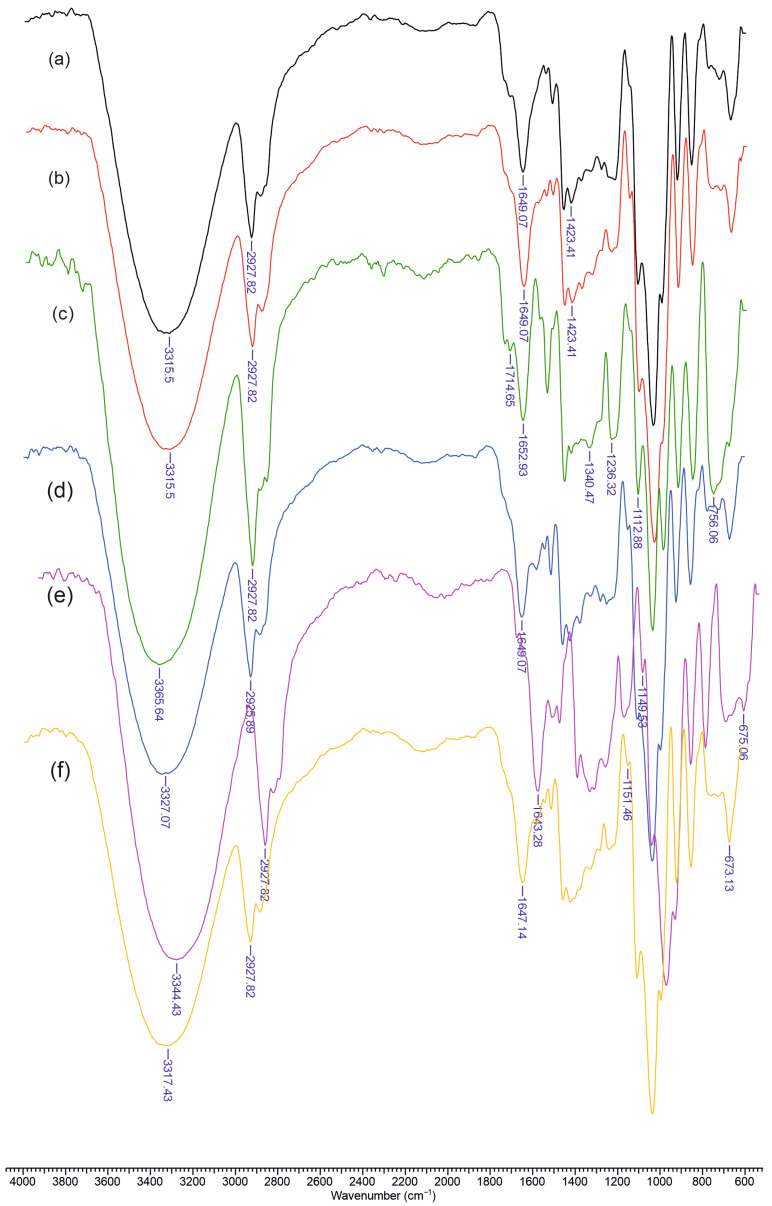
FTIR spectra of the obtained materials: (**a**) Chi-L; (**b**) Chi-Dex-L; (**c**) Chi-Dex-L-Cu; (**d**) Chi-L-Cu; (**e**) Chi-mDex-L; (**f**) Chi-mDex-L-Cu.

Figure 2 presents the FTIR spectra of the materials containing chitosan (Chi), dextran (Dex) or carboxymethyl dextran (mDex), lignin (L), and curcumin (Cu). In spectrum (a), corresponding to Chi–L, a broad absorption centered around 3300–3400 cm^−1^ is evident, reflecting overlapping O–H (from lignin phenolic groups) and N–H (from chitosan) stretching bands. A moderate peak near 2920 cm^−1^ is attributed to C–H stretching vibrations, common to polysaccharides and lignin’s aliphatic side chains. The amide I region of chitosan (around 1640–1650 cm^−1^) and the aromatic ring vibrations of lignin (approximately 1600 and 1510 cm^−1^) overlap slightly, although the lignin band near 1510 cm^−1^ remains visible. Chitosan’s saccharide ring vibrations appear in the 1150–1000 cm^−1^ region, producing a characteristic polysaccharide fingerprint.

Upon addition of dextran, as shown in spectrum (b) (Chi–Dex–L), the broad hydrogen-bonded region (3300–3400 cm^−1^) remains prominent, but the 1150–1000 cm^−1^ region becomes more intense due to the glycosidic ring signals of dextran. The lignin aromatic ring vibrations and chitosan amide band persist near 1600 and 1650 cm^−1^, respectively. Introducing curcumin into the Chi–Dex–L matrix—spectrum (c) (Chi–Dex–L–Cu)—adds a conjugated C=O stretching band (1620–1630 cm^−1^) that overlaps with the lignin/chitosan region. Additional aromatic ring modes from curcumin overlap with those of lignin near 1500–1450 cm^−1^, and the phenolic O–H of curcumin contributes further to broadening in the 3300–3400 cm^−1^ range.

A similar trend appears in spectrum (d) (Chi–L–Cu), where the absence of dextran simplifies the carbohydrate region, yet the curcumin band near 1620–1630 cm^−1^ and lignin aromatic absorptions around 1600 and 1510 cm^−1^ remain clearly visible. In spectrum (e) (Chi–mDex–L), the carboxylate groups in carboxymethyl dextran shift or intensify absorptions near 1600–1610 cm^−1^. Finally, in spectrum (f) (Chi–mDex–L–Cu), the characteristic signals of all four components—chitosan, mDex, lignin, and curcumin—overlap. The broad band at 3300–3400 cm^−1^ encompasses O–H and N–H stretching from all components, while the COO^−^ stretch (mDex), the aromatic ring vibrations (lignin and curcumin), and the saccharide skeleton bands (chitosan and mDex) combine, confirming effective integration of each constituent into the material.

### 2.2. Mechanical Properties of Materials

**Figure 3:**
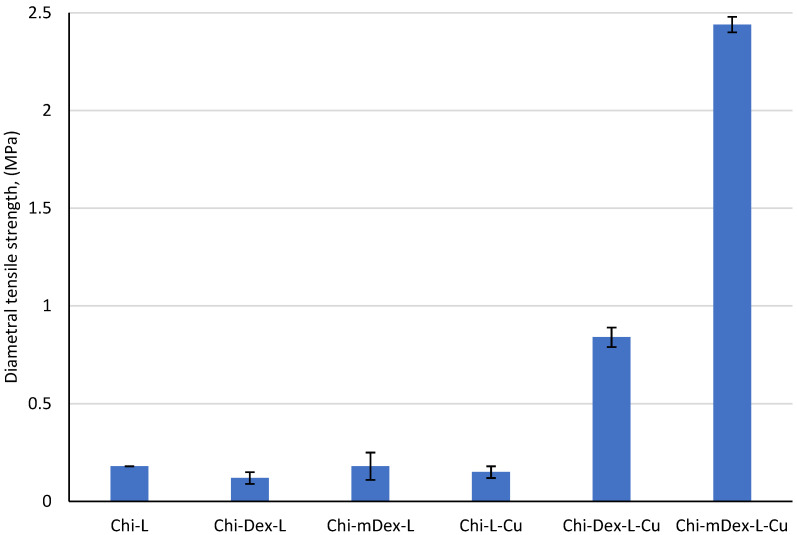
Diametral tensile strength for tested materials.

The diametral tensile strength (DTS) data (Figure 3) reveal clear material’s composition–property correlations within these formulations. Chi–L exhibits a moderate DTS value of 0.18 MPa, reflecting hydrogen bonding and limited electrostatic interactions between chitosan’s amine groups and lignin’s phenolic moieties. Addition of dextran (Chi–Dex–L) diminishes DTS to 0.12 MPa—a 33% decrease—likely because dextran’s relatively linear structure and hydroxyl groups do not contribute enough additional crosslinking sites to strengthen the matrix. In contrast, replacing dextran with carboxymethyl dextran (Chi–mDex–L) recovers the original 0.18 MPa, indicating that the carboxymethyl groups facilitate somewhat stronger hydrogen bonding or ionic interactions that restore the baseline strength.

The introduction of curcumin (Cu) drives dramatic enhancement in the DTS of certain formulations. Although Chi–L–Cu (0.15 MPa) is 17% lower than the original Chi–L, adding curcumin to Chi–Dex–L (Chi–Dex–L–Cu) boosts the DTS to 0.84 MPa—an increase of 600% over its non-curcumin counterpart, and nearly 370% higher than Chi–L. Even more striking is the Chi–mDex–L–Cu, achieving 2.40 MPa: a 1233% increase relative to Chi–mDex–L and 1200% higher than Chi–L. These improvements reflect the multifunctional role of curcumin, whose polyphenolic rings can participate in hydrogen bonding, hydrophobic interactions [40], and potential electrostatic attractions with both chitosan and lignin. Furthermore, the carboxyl groups of mDex introduce additional binding sites that amplify curcumin’s crosslinking efficacy, leading to highly interconnected, mechanically robust networks. Hence, the synergistic effect of curcumin and mDex produces the most significant rising in DTS, underscoring the importance of complementary functional groups for maximizing material strength.

The diametral tensile strength of the tested materials exhibited significant variations, as confirmed by the ANOVA results (F = 936.1433, *p* = 4.09 × 10^−15^), indicating that the mechanical properties are strongly influenced by the material composition and the presence of curcumin (Table S1, Supplementary Materials). The post-hoc Bonferroni test further confirmed significant differences between specific groups, particularly between curcumin-loaded and non-loaded materials.

Among the non-curcumin-loaded materials, Chi-L (0.18 MPa), Chi-Dex-L (0.12 MPa), and Chi-mDex-L (0.18 MPa) exhibited similar tensile strengths, with no statistically significant differences between them (*p* > 0.05). The introduction of curcumin had a profound impact on the mechanical properties, as evidenced by the significant augmentation in DTS values for Chi-Dex-L-Cu (0.84 MPa) and Chi-mDex-L-Cu (2.44 MPa). The tensile strength of Chi-mDex-L-Cu was significantly higher than all other groups (*p* < 3.4 × 10^−7^ when compared to Chi-L, Chi-Dex-L, and Chi-L-Cu).

While Chi-L-Cu (0.15 MPa) did not show a significant rise in strength compared to its non-curcumin counterpart (Chi-L, *p* = 0.607), the system comprising modified dextran exhibited a distinct mechanical enhancement when curcumin was incorporated. Chi-Dex-L-Cu showed a nearly sevenfold increase in strength compared to Chi-Dex-L (*p* = 6.31 × 10^−5^), and Chi-mDex-L-Cu demonstrated an even more pronounced increase (*p* = 3.23 × 10^−7^). These results suggest that the interaction between curcumin and Chi-Dex-based matrices contributes to enhanced mechanical reinforcement, likely due to improved structural integrity and polymer cross-linking effects.

The findings demonstrate that Chi-mDex-L-Cu is the most mechanically robust material, exhibiting the highest diametral tensile strength among all tested formulations. This enhanced mechanical performance makes it a promising candidate for applications requiring structural stability and durability, such as biomedical scaffolds or drug delivery systems requiring mechanical resilience. In contrast, Chi-L-Cu exhibited only marginal improvements in mechanical properties, suggesting that the chitosan-lignin system alone does not provide significant reinforcement upon curcumin loading.

Overall, the results emphasize that the mechanical behavior of curcumin-loaded materials depends not only on the presence of curcumin but also on the specific polymeric matrix used. The formulations comprising dextran, particularly Chi-mDex-L-Cu, benefit from a synergistic effect that significantly enhances their tensile strength, making them more suitable for applications requiring both controlled drug release and improved mechanical stability.

### 2.3. Scanning Electron Micrography

**Figure 4:**
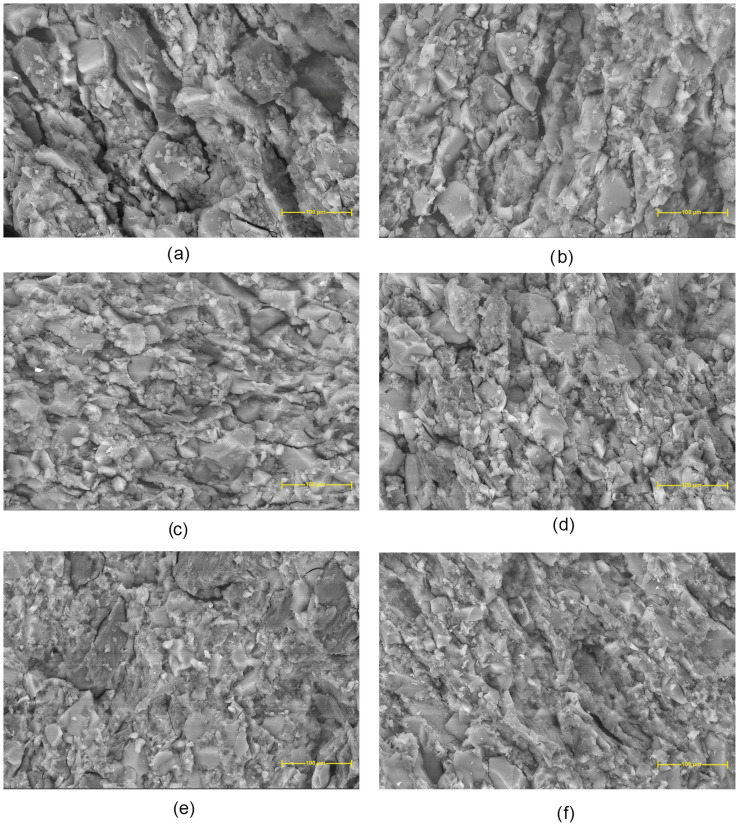
Scanning electron micrographs of materials: (**a**) Chi-L; (**b**) Chi-Dex-L; (**c**) Chi-mDex-L; (**d**) Chi-L-Cu; (**e**) Chi-Dex-L-Cu; (**f**) Chi-mDex-L-Cu.

Scanning electron micrographs (Figure 4) of Chi–L reveal a relatively coarse, granular surface with discernible pores and micro voids, reflecting characteristic chitosan–lignin aggregation. In Chi–Dex–L, the overall architecture appears somewhat denser, and while pores are still visible, they seem smaller or less numerous, suggesting that dextran may enhance interfacial cohesion and partially fill void spaces. By contrast, Chi–mDex–L exhibits more uniform domains and fewer apparent pores, consistent with additional electrostatic or hydrogen bonding introduced by the carboxymethyl groups, which may further reduce or mask void regions.

Upon curcumin loading, Chi–L–Cu shows granular deposits interspersed with remnant pores, indicating partial filling of cavities by curcumin. In Chi–Dex–L–Cu, curcumin similarly appears to occupy some of the existing pore space, leading to a slightly more compact matrix. Finally, Chi–mDex–L–Cu contains fewer visible voids, suggesting that the synergistic effect of carboxymethyl dextran and curcumin results in a more uniformly filled structure with minimized pore dimensions. Overall, these micrographs underscore how each component modulates pore formation and surface compactness in the chitosan-dextran based materials.

### 2.4. Release Kinetic of Bioactive Principles from Polymeric Matrix

The Weibull model is a versatile mathematical tool widely employed to describe the kinetics of drug release from polymeric matrices. Its flexible form allows for the characterization of various release mechanisms, ranging from diffusion-dominated processes to degradation-controlled systems. The general equation (Equation (1)) of the Weibull model is:

(1) $$y=A\times \left(1-e^{-{\left(k\times \left(x-x_{c}\right)\right)}^{d}}\right)$$

where: *y*—cumulative drug release at time *x*; *A*—maximum cumulative release (asymptotic value); *k*—release rate constant, indicating the speed of drug release; *x_c_*—lag time before significant drug release begins; *d*—shape parameter, which determines the nature of the release curve.

By fitting experimental data to the Weibull model, the values of *A*, *k*, *x_c_*, and *d* provide valuable insights into the drug release profile: *A*—indicates the total amount of drug that can be released; *k*—reflects the speed of release, influenced by the matrix’s structure and drug properties; *x_c_*—identifies any delay in release, such as a hydration phase or lag due to matrix swelling; *d*—reveals the dominant release mechanism, distinguishing between diffusion and matrix degradation.

The model accommodates various types of polymeric matrices, including hydrogels, crosslinked networks, and composite materials. Its adaptability allows it to describe systems with single or combined release mechanisms.

The Weibull model is often used to fit experimental release data, enabling the determination of the kinetics and mechanisms of drug release. For example, a low *d* value (<1) indicates diffusion-dominated release, common in hydrophilic polymeric matrices.

By comparing *k* and *d* values across different formulations, researchers can evaluate the influence of polymer composition, crosslinking density, and drug-polymer interactions on release behavior.

The model assists in designing formulations tailored for specific therapeutic needs. For instance, fast-release systems can be designed by optimizing matrices with higher *k* and *A* values. Sustained-release systems can be achieved by introducing carboxylic groups or enhancing crosslinking, reducing *A* and *k*.

The Weibull model is a cornerstone in the study of drug release kinetics from polymeric matrices. Its flexibility and interpretability make it invaluable for understanding and optimizing release profiles. By using the insights provided by the model, there could be designed advanced drug delivery systems adapted for a wide range of therapeutic applications. Whether for fast-acting formulations or prolonged-release therapies, the Weibull model remains a powerful tool in pharmaceutical research.

**Figure 5:**
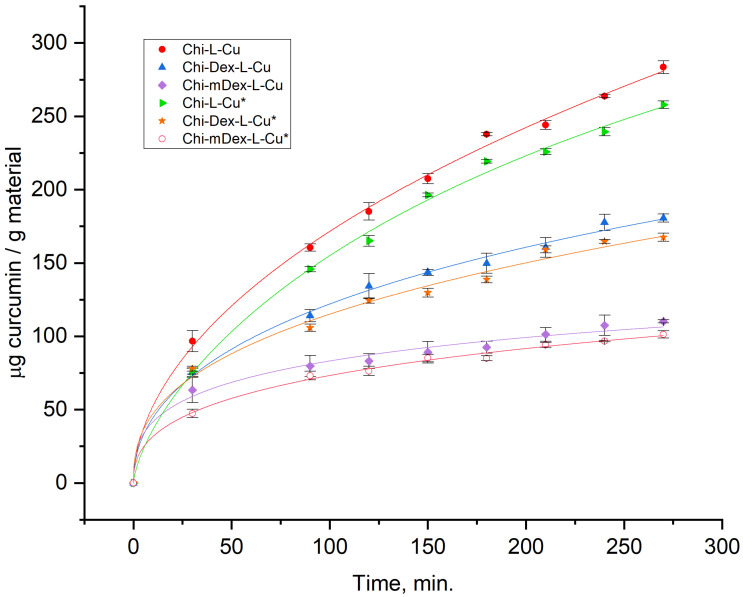
Curcumin release from tested materials.

The release of curcumin from Chi-L-Cu, Chi-Dex-L-Cu, Chi-mDex-L-Cu, Chi-L-Cu*, Chi-Dex-L-Cu*, and Chi-mDex-L-Cu* materials was assessed using the Weibull model to evidence the release kinetics (Figure 5). The parameters analyzed include the maximum release (*A*) and the rate constant (*k*), which are influenced by the composition and chemical structure of the materials components (Table 1).

The release kinetics of pure curcumin (Cu*) from the chitosan-dextran/carboxymethyl dextran-lignin matrix exhibited a significantly slower and lower cumulative release compared to curcumin extracted from turmeric. This behavior can be attributed to the strong molecular interactions between curcumin and the polymeric components, including hydrogen bonding, electrostatic interactions, and π-π stacking with lignin. These interactions contribute to curcumin entrapment within the polymeric network, limiting its diffusion and prolonging its release. Additionally, the polymeric matrix forms a semi-rigid, hydrophilic environment that restricts the solubility and mobility of curcumin, further reducing the release rate. In contrast, curcumin in turmeric extract exists in a more bioavailable form, likely due to its association with other bioactive compounds that enhance its solubility and diffusion.

Chi-L-Cu exhibited the highest release capacity, with an asymptotic *A* value of 400 µg. This represents a 3.47% higher release compared to Chi-Dex-L-Cu (*A* = 386) and a 55.66% higher release as compared to Chi-mDex-L-Cu (*A* = 177.37). The significantly lower A value for Chi-mDex-L-Cu suggests that the introduction of carboxymethyl dextran into the matrix substantially limits curcumin release. The carboxymethylation process introduces carboxylic (-COOH) groups, which enhance crosslinking density, thereby reducing matrix porosity and curcumin diffusion.

Chi-L-Cu demonstrated the fastest release rate, with *k* = 0.00456. This was 68.16% higher than Chi-Dex-L-Cu (*k* = 0.00145) and 35.96% higher than Chi-mDex-L-Cu (*k* = 0.00292). The slower k recorded for Chi-Dex-L-Cu reflects the impact of dextran incorporation, which increases matrix rigidity, slowing the diffusion of curcumin. Chi-mDex-L-Cu, with its lower *k*, balances curcumin diffusion and matrix degradation. The presence of carboxylic groups in carboxymethyl dextran enhances ionic interactions with chitosan’s amine groups, creating a compact and highly crosslinked network.

Carboxymethyl dextran contains carboxyl (-COOH) groups that significantly influence the matrix structure. These groups react with the amine (-NH_2_) groups in chitosan, forming ionic crosslinks that increase matrix density. Hydrogen bonding between carboxylic groups and hydroxyl groups in lignin or dextran further strengthens the network, enhancing mechanical stability. The additional crosslinking reduces water uptake and matrix swelling, thereby limiting the diffusion of curcumin. To conclude, the chemical modification of dextran (carboxymethylation) introduces additional reactive groups, which: increase the potential for crosslinking within the matrix; reduce permeability of the material, leading to lower *A* and slower *k*; in Chi-mDex-L-Cu, the enhanced crosslinking results in a highly compact structure, making it ideal for applications requiring prolonged and controlled release.

The differences in *A* and *k* between the materials highlight the impact of polymeric composition and chemical modifications on curcumin release: Chi-L-Cu—high *A* and *k*, indicating a permeable matrix ideal for rapid and extensive curcumin release; Chi-Dex-L-Cu—moderate *A* and *k*, reflecting the effect of dextran incorporation, which slows release while maintaining a high release capacity; Chi-mDex-L-Cu—the lowest *A* and lowest *k*, underlining the role of carboxymethyl dextran in creating a denser, more rigid matrix with prolonged release.

These findings suggest that the chemical modifications of dextran, particularly the introduction of carboxylic groups, play a pivotal role in optimizing the release profiles of curcumin, enabling the design of materials for specific drug delivery applications.

The controlled release of curcumin from polymeric matrices was analyzed through key pharmacokinetic parameters, including area under the curve (AUC), maximum drug concentration (C_max_), and time to maximum concentration (T_max_). These parameters provide insight into the extent, rate, and duration of drug release from the tested materials: Chi-L-Cu, Chi-Dex-L-Cu, and Chi-mDex-L-Cu.

The computed AUC values were 43,950.0, 29,425.0, and 20,025.0 µg·min/g material for Chi-L-Cu, Chi-Dex-L-Cu, and Chi-mDex-L-Cu, respectively. The corresponding C_max_ values were 250.0, 165.0, and 110.0 µg/g material, all occurring at T_max_ of 270 min (Table S2, Supplementary Materials). These results indicate distinct drug release behaviors across the three formulations.

Chi-L-Cu exhibited the highest AUC, suggesting the greatest total drug exposure over time. The peak concentration of 250.0 µg/g material at 270 min confirms that this formulation achieved the highest release among the tested materials. The combination of a high AUC and Cmax indicates a rapid and efficient drug release, likely attributed to a porous polymeric matrix that facilitates curcumin diffusion. This system is optimal for applications requiring high initial drug availability, where curcumin must reach therapeutic levels quickly.

Chi-Dex-L-Cu demonstrated an AUC of 29,425.0 µg·min/g material, lower than Chi-L-Cu but still significant. The C_max_ of 165.0 µg/g material at 270 min suggests a more controlled release compared to Chi-L-Cu. The presence of dextran within the chitosan matrix likely contributes to a reduction in diffusion rates, promoting sustained release characteristics. This formulation is suitable for applications requiring extended drug delivery, where maintaining moderate curcumin concentrations over time is beneficial.

Chi-mDex-L-Cu exhibited the lowest AUC of 20,025.0 µg·min/g material, indicating the least overall drug exposure. The C_max_ of 110.0 µg/g material at 270 min reflects the slowest drug release among the three formulations. The modified dextran component appears to further restrict curcumin diffusion, leading to a gradual and prolonged release profile. This formulation is ideal for long-term drug administration, where maintaining a lower but consistent drug concentration over time is required.

A comparative analysis of the three formulations suggests that material composition significantly influences the release kinetics of curcumin. Chi-L-Cu is best suited for rapid drug delivery, ensuring high curcumin availability early in the release period. Chi-Dex-L-Cu offers a balance between rapid and sustained release, making it an effective controlled-release system. Chi-mDex-L-Cu provides the most prolonged release profile, maintaining curcumin concentrations at lower but steady levels, making it ideal for applications requiring long-term therapeutic effects. These findings highlight the potential for tailoring polymeric drug delivery systems on specific therapeutic requirements.

The delivery of curcumin from the three tested polymeric materials (Chi-L-Cu, Chi-Dex-L-Cu, and Chi-mDex-L-Cu) exhibit significant differences, as shown in the release profile (Figure 5). The release trends indicate that Chi-L-Cu presents the highest release rate and maximum cumulative release, followed by Chi-Dex-L-Cu, while Chi-mDex-L-Cu shows the lowest release of curcumin over time.

The ANOVA results (Table S3, Supplementary Materials) confirm a statistically significant difference between the release profiles of the tested materials (F = 1633.561, *p* = 6.16 × 10^−9^), indicating that the variations observed in curcumin release are not due to random fluctuations but are attributable to differences in materials composition. The post-hoc Bonferroni correction further validates these findings, with all pairwise comparisons being statistically significant (*p* < 0.0003 in all cases).

Chi-L-Cu exhibited the highest release rate and maximum cumulative release, making it ideal for fast-release applications, such as immediate drug delivery where rapid bioavailability is required.

Chi-Dex-L-Cu demonstrated a more moderate and sustained release profile, which could be beneficial for controlled-release formulations, where a prolonged therapeutic effect is desired while minimizing drug fluctuations.

Chi-mDex-L-Cu displayed the slowest and lowest release, suggesting its suitability for extended-release or long-term drug delivery systems, where a gradual and sustained drug release is required to reduce dose administration.

These findings emphasize the influence of polymeric modifications on the drug release behavior, highlighting the potential of chitosan-dextran based matrices for adjusting drug delivery to specific therapeutic needs.

### 2.5. Anti-Inflammatory Activity of Materials

BSA (bovine serum albumin) protein denaturation assay for the in vitro evaluation of anti-inflammatory potential of curcumin circumvented the ethical issues associated with the use of animals, having in mind that these are results of the first screenings.

**Figure 6:**
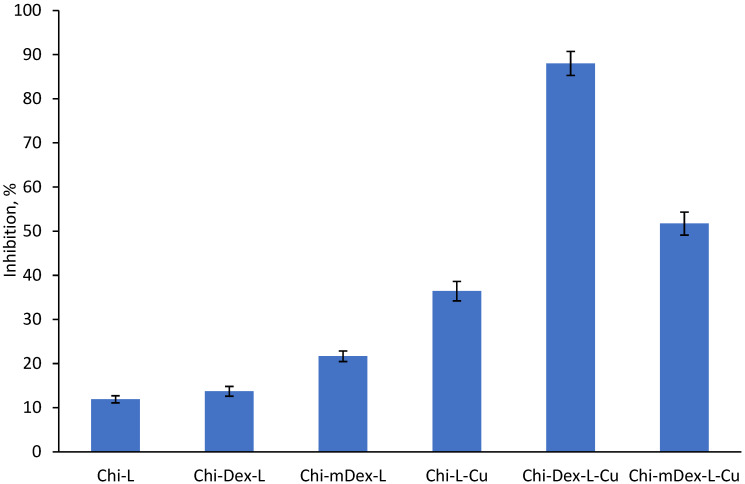
Anti-inflammatory activity of materials.

The curcumin release profiles from the tested materials (Figure 5) reveal distinct kinetic behaviors that significantly influence their anti-inflammatory efficacy (Figure 6). Among the formulations, Chi-L-Cu exhibits the fastest curcumin release, followed by Chi-Dex-L-Cu, while Chi-mDex-L-Cu demonstrates the most sustained and controlled release over time. This suggests that the nature of the polymer matrix plays a crucial role in curcumin diffusion, with the carboxymethyl dextran (mDex) component contributing to prolonged retention of curcumin within the polymeric network. The presence of carboxyl groups in mDex likely enhances hydrogen bonding and electrostatic interactions, slowing down the diffusion rate while maintaining a continuous, steady release.

The anti-inflammatory data further support the idea that curcumin bioavailability is strongly linked to its release kinetics. Chi-Dex-L-Cu achieves the highest inhibition (~90%), demonstrating that an intermediate release rate optimally balances bioavailability with sustained action. Notably, Chi-mDex-L-Cu exhibits substantial anti-inflammatory activity (~70%) despite slower curcumin release, indicating that its controlled release mechanism may offer prolonged therapeutic effects, potentially reducing the need for frequent dosing. This is a key advantage in biomedical applications where sustained drug release is desirable for long-term anti-inflammatory effects.

While Chi-L-Cu releases curcumin more rapidly, leading to an initial strong inhibition effect, its faster depletion may limit prolonged bioactivity. In contrast, Chi-mDex-L-Cu, with its extended-release profile, could be of particular interest for applications requiring prolonged curcumin action, such as chronic inflammatory conditions. The ability of mDex to modulate curcumin release while still achieving significant inhibition underscores its potential as a strategic polymeric component in sustained-release formulations.

Overall, these results highlight that mDex-containing formulations not only ensure extended curcumin availability but also retain significant anti-inflammatory efficacy, making them highly attractive for long-term therapeutic applications. Future studies should explore further optimization of mDex-based matrices to fine-tune release rates and to enhance sustained bioactivity for applications in osteoarthritis treatment, or gastrointestinal inflammatory disorders.

The release kinetics of curcumin from the tested materials exhibit distinct profiles, which directly impact their anti-inflammatory effectiveness. The release profile demonstrated that Chi-L-Cu exhibited the highest curcumin release rate and cumulative release over time, followed by Chi-Dex-L-Cu, while Chi-mDex-L-Cu displayed the slowest and lowest curcumin release. The statistical analysis of anti-inflammatory capacity (Table S4, Supplementary Materials) further confirmed significant differences among the tested materials, as indicated by the ANOVA results (F = 456.308, *p* = 2.99 × 10^−13^). The post-hoc Bonferroni test revealed that nearly all compared pairwise were statistically significant (*p* < 0.0003 in most cases), reinforcing the impact of dextran modifications on curcumin bioavailability and therapeutic efficiency.

Chi-L-Cu exhibited an average anti-inflammatory activity of 36.42%, correlating with its rapid curcumin release, suggesting that materials with high initial release provide quick bioavailability and an early anti-inflammatory effect. Chi-Dex-L-Cu showed the highest overall anti-inflammatory activity, with an average of 88.01%, aligning with its more controlled release profile. This suggests that a moderate and sustained release enhances prolonged bioactivity, preventing the rapid depletion of curcumin and thereby maintaining an extended therapeutic effect. Chi-mDex-L-Cu, while exhibiting the lowest release profile, displayed significant anti-inflammatory activity with an average of 51.73%, higher than Chi-L-Cu but lower than Chi-Dex-L-Cu. This indicates that while the slow release profile may delay maximum effectiveness, it still provides prolonged anti-inflammatory action.

These findings demonstrate a strong correlation between curcumin release kinetics and its biological activity. Fast-releasing materials such as Chi-L-Cu are suitable for applications requiring immediate anti-inflammatory action, whereas controlled-release systems like Chi-Dex-L-Cu are more effective for prolonged therapeutic effects. Slow-release systems, such as Chi-mDex-L-Cu, may be advantageous for extended treatments where a sustained, lower-dose effect is required. The results highlight the importance of dextran modifications in optimizing curcumin delivery for enhanced bioactivity and suggest that carefully tuning release kinetics is essential for maximizing therapeutic benefits in drug delivery applications.

### 2.6. Antioxidant Activity of Materials

**Figure 7:**
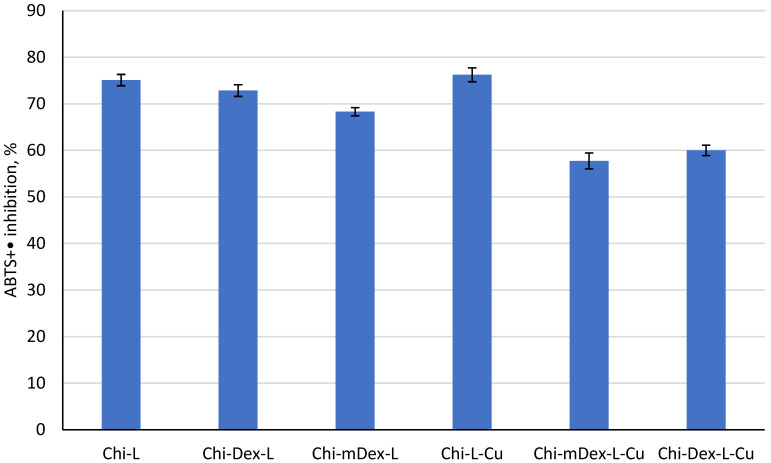
Antioxidant capacity of the tested materials.

The antioxidant activity of the tested materials, as evaluated using the ABTS assay (Figure 7), highlights the influence of both the polymeric matrix and curcumin release kinetics on radical scavenging capacity. When fillers were incorporated into the matrix, the antioxidant properties were modified, with Chi-Dex-L showing a similar inhibition rate (~72%), while the inclusion of carboxymethyl dextran (mDex) slightly reduced the antioxidant activity to ~68%.

Curcumin, known for its potent inhibitory capacity, plays a crucial role in modulating the antioxidant activity of these materials. Its C=C bonds, β-diketo group, and phenyl rings contribute significantly to radical scavenging, acting as H-atom donors in antioxidant mechanisms [41]. This effect is evident in the Chi-L-Cu (~80% inhibition) and Chi-Dex-L-Cu (~65% inhibition) samples, which exhibited the highest antioxidant activity due to the presence of phenolic hydroxyl groups in both curcumin and lignin, capable of donating hydrogen protons to neutralize free radicals [42]. However, the most intriguing finding is that while Chi-mDex-L-Cu demonstrated slightly lower radical scavenging capacity (~60%), its extended curcumin release profile (Figure 5) suggests a prolonged antioxidant effect over time.

Unlike Chi-L-Cu, which releases curcumin rapidly and reaches higher radical scavenging activity in the short term, Chi-mDex-L-Cu ensures a more sustained release of curcumin, maintaining antioxidant potential over an extended period. This suggests that while its immediate inhibition rate is ~25% lower than Chi-L-Cu, it may offer prolonged antioxidant protection, making it a lead candidate for applications where sustained activity is required, such as food packaging, biomedical coatings, or pharmaceutical formulations for chronic oxidative stress conditions.

Overall, these results emphasize the strategic role of mDex-based matrices in modulating curcumin release and prolonging its bioactivity. Future research should explore optimizing these formulations to fine-tune the balance between initial radical scavenging capacity and sustained curcumin availability, positioning Chi-mDex-L-Cu as a promising material for long-term antioxidant applications.

The relationship between curcumin release and antioxidant activity plays a crucial role in evaluating the effectiveness of polymers carriers for bioactive compound delivery. The release kinetics demonstrated that Chi-Dex-L-Cu and Chi-mDex-L-Cu exhibited a more controlled release profile compared to Chi-L-Cu, which showed the highest and fastest curcumin release. This controlled release behavior is particularly relevant when considering the antioxidant efficiency over time, as the antioxidant activity is directly dependent on the amount of curcumin available at a given time (t).

The statistical analysis of antioxidant capacity confirmed significant differences among the tested materials, with ANOVA results (Table S5, Supplementary Materials) indicating a highly significant effect (F = 73.01071, *p* = 1.46 × 10^−8^). Post-hoc Bonferroni analysis showed that while Chi-L-Cu demonstrated the highest initial antioxidant capacity, Chi-Dex-L-Cu and Chi-mDex-L-Cu exhibited a more sustained antioxidant effect, attributed to the controlled curcumin release. The average antioxidant activity for Chi-Dex-L-Cu (57.74%) and Chi-mDex-L-Cu (60.00%) was lower than that of Chi-L-Cu (76.23%); however, this difference must be interpreted in the context of release kinetics. At a given time point, the antioxidant capacity is proportional to the cumulative amount of curcumin released, meaning that a slower but continuous release, as observed in Chi-Dex-L-Cu and Chi-mDex-L-Cu, ensures prolonged bioactivity.

A key observation is that while Chi-L-Cu provides an initial burst of antioxidant activity due to rapid curcumin release, its efficiency may decline over time as the available curcumin is depleted. In contrast, Chi-Dex-L-Cu and Chi-mDex-L-Cu maintain a more stable release profile, ensuring that curcumin remains bioavailable over extended periods, thus sustaining antioxidant activity at later time points. This controlled-release mechanism is particularly advantageous in applications where long-term free radical scavenging is required rather than an immediate but transient effect. The statistical significance of the differences between materials further supports this finding, with Chi-Dex-L-Cu and Chi-mDex-L-Cu demonstrating distinct antioxidant behaviors compared to Chi-L-Cu (*p* < 0.0006 in most cases).

These results highlight that Chi-Dex-L-Cu and Chi-mDex-L-Cu are particularly valuable for applications requiring sustained antioxidant activity, such as chronic oxidative stress management or prolonged therapeutic delivery. Their ability to regulate curcumin availability over time enhances their potential for biomedical applications, where maintaining a steady antioxidant effect is more beneficial than a rapid but short-lived response. The findings emphasize the importance of selecting matrices components to optimize curcumin bioavailability and maximize its therapeutic potential over extended durations.

### 2.7. In Vitro Biocompatibility of Samples

The proliferative effect of the developed materials was evaluated using the MTS (3-[4,5-dimethylthiazol-2-yl]-2,5-diphenyl tetrazolium bromide) assay against gingival fibroblasts. The method is a widely used, reliable colorimetric method to assess cell viability and cytotoxicity, based on the ability of mitochondrial dehydrogenases in metabolically active cells to reduce MTS into formazan crystals. The amount of formazan produced is directly proportional to the number of viable cells, providing a quantitative measure of cell survival and proliferation.

**Figure 8:**
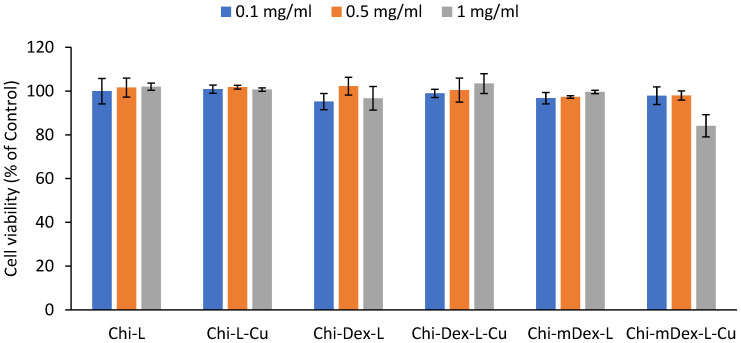
In vitro biocompatibility of samples.

After 24 h of incubation, all tested formulations exhibited high cell viability across the three concentrations (0.1 mg/mL, 0.5 mg/mL, and 1 mg/mL), confirming their excellent biocompatibility (Figure 8). The cell survival rates remained above 85% in all cases, indicating low cytotoxicity and minimal interference with cellular metabolic activity.

Among the tested materials, Chi-L and Chi-L-Cu demonstrated the highest cell viability (~95–100%), suggesting that the chitosan-lignin does not induce adverse effects on fibroblast proliferation. Similarly, Chi-Dex-L and Chi-Dex-L-Cu maintained high biocompatibility, with cell viability exceeding 90% across all tested concentrations, indicating that the incorporation of dextran and curcumin does not negatively impact cell survival and further supports their potential as safe therapeutic materials.

Chi-mDex-L and Chi-mDex-L-Cu also displayed excellent biocompatibility, although a slight decrease in viability (~10% reduction at 1 mg/mL for Chi-mDex-L-Cu) was observed compared to other formulations. This reduction is likely attributed to the increased interaction between carboxymethyl dextran (mDex) and the fibroblast membrane at higher concentrations. However, even at the highest tested dose, cell viability remained above 80%, reinforcing the safety profile of mDex-containing formulations.

For an MTS assay, cell viability above 80% is generally considered non-cytotoxic, while viability between 60–80% suggests moderate biocompatibility and below 60% may indicate cytotoxic effects. In this study, all materials met the biocompatibility criteria, confirming their potential for biomedical applications.

Given their biocompatibility and potential for sustained curcumin release, mDex-based formulations stand out as lead candidates for in vivo evaluation, where their long-term therapeutic efficacy and biological interactions will be further assessed.

## 3. Materials and Methods

### 3.1. Materials

Dextran from *Leuconostoc* spp. (100,000 Da), chitosan from shrimp shells (250,000 Da, viscosity 800 cps, purity 98.7% and degree of deacetylation of 83.28%), curcumin (Cu*, with a purity ≥ 94%, cat. no. C7727), monochloroacetic acid (99%), glutaraldehyde solution (50 wt.% in H_2_O), 4,5-Dihydroxynaphthalene-2,7-disulfonic acid disodium salt (chromotropic acid, reagent grade > 98.5%), bovine albumin (reagent grade > 97%) and 2,2′-Azino-bis(3-ethylbenzothiazoline-6-sulfonic acid (>98%) (ABTS) were from Sigma-Aldrich Co. (St. Louis, MO, USA) and were used without any further purification. The softwood lignin (Lignoboost softwood) was obtained at Södra Cell (Växjö, Sweden), Sweden by regular Lignoboost process.

### 3.2. Extraction of Curcumin (Cu)

A total of 20 g of finely powdered turmeric was subjected to solvent extraction using 100 mL of 95% ethanol in a 250 mL beaker. The mixture was stirred at 40 °C for 2 h, ensuring efficient solubilization of curcuminoids. The resulting suspension was filtered, and the filtrate was concentrated under reduced pressure at 50 °C using a rotary evaporator. To induce curcumin precipitation, 50 mL of cold distilled water (4 °C) was slowly added to the concentrated extract under gentle stirring. The mixture was then left undisturbed at 4 °C for 12 h to allow complete crystallization. The precipitated curcumin was collected by vacuum filtration, washed with hexane and cold ethanol, and dried in a vacuum oven at 40 °C for 24 h. The final yield of purified curcumin was 2.9376 g, corresponding to an extraction efficiency of 14.69% (*w*/*w*) relative to the initial turmeric powder.

### 3.3. Synthesis of Carboxymethyl Dextran (mDex)

2 g of dextran were dissolved in 150 mL of 2.0 M NaOH solution. Then, 30 mL of chloroacetic acid of concentration of 0.6% was added. After stirring at room temperature for 24 h, the mixtures were dialyzed against deionized water for 1 week. The white powder of carboxymethyl dextran obtained after lyophilization was used as matrix component.

### 3.4. The Obtainment of Materials

The materials were obtained via blending method. A Chi solution was prepared by dissolving 0.75 g Chi in 75 mL (1 % *v*/*v*) acetic acid aqueous solution, stirring thoroughly for 1 h. Also, solution of 0.1 g of dextran/ modified dextran in water was prepared. Cu/Cu* (0.1 g) was dissolved in ethanol (5 mL) and mixed thoroughly ensure uniform dispersion in each previously obtained solution. The obtained formulations are noted in Table 2. Furthermore, 100 µL of glutaraldehyde was added to every formulation, which was stirred at 40 °C for few seconds to obtain a material-forming solution. Each material was poured into a 90 mm diameter Petri dish and left to dry for 48 h. After casting, capsules (named according to Table 2) with an 11 mm diameter and 1 mm thickness by using a Carver Hydraulic Laboratory Press Model C at a pressure of 6 tons for 2 min were obtained.

### 3.5. The Determination of Degree of Substitution

The degree of substitution (DS) of the carboxymethyl dextran (mDex) sample was quantified following the approach outlined by Li et al. [43], with minor alterations. Briefly, 100 mg of glycolic acid was dissolved in 10 mL of distilled water and brought to neutrality by adding 250 mmol/L NaOH. Next, calibration standards covering 0–500 μg/mL of glycolic acid were prepared, each combined with 5 mL of a 1 g/L chromotropic acid solution and 1 mL of concentrated sulfuric acid in colorimetric tubes. These mixtures were then placed in a boiling water bath for 30 min, cooled to room temperature, and diluted to 25 mL using a 30% ammonium acetate solution. Their absorbances were recorded at 570 nm with a Jenway 6405 UV-Vis spectrophotometer (Cole-Parmer, Vernon Hills, IL 60061, USA), generating the standard calibration curve. A separate aliquot of mDex (0.5 mg) dissolved in 0.5 mL distilled water underwent the same procedure. The *DS* value was subsequently calculated according to the relationship (Equation (2)):

(2) $$DS=\frac{162\times A}{\left(76-58\times A\right)}$$

where *A* represents the mass of glycolic acid (in grams) per gram of mDex sample, 76 g/mol is the molecular weight of glycolic acid, and 58 g/mol is the molecular weight corresponding to the substituted carboxymethyl moiety. Determination of *DS* was performed in triplicate. The synthesized mDex presented a degree of substitution *DS* = 0.92 ± 0.1123.

### 3.6. Fourier Transform Infrared (FTIR) Spectroscopy

FT-IR spectra of all materials were recorded using a Vertex 70 FTIR spectrometer from Brüker (Billerica, MA, USA), equipped with an ATR (Attenuated Total Reflectance) device with a ZnSe crystal. Maintaining a spectral resolution of 2 cm^−1^, measurements were carried out at a 45-degree angle of incidence, covering the range of 4000–600 cm^−1^.

### 3.7. Mechanical Properties

The mechanical properties were evaluated using a universal testing machine (Shimadzu AG-IC, Kyoto, Japan), with a loading rate of 0.5 mm per minute [44]. The mechanical properties of the materials were tested on tablets with an 11 mm diameter and 3 mm thickness compressed using a Carver Hydraulic Laboratory Press (Model C) under a pressure of 6 tons for 2 min. The compression was conducted before analysis to ensure uniformity in sample preparation. The compression was conducted before analysis to ensure uniformity in sample preparation. The collected load-deflection curves were used to determine the maximum compression load at failure. The provided value was the mean of five measurements. Experiments ware performed in triplicate.

### 3.8. Scanning Electron Microscopy (SEM)

The materials’ morphologies were investigated by scanning electron microscope (SEM). The SEM analysis was conducted using a QANTA FEG 250 Scanning Electron Microscope. The applied acceleration voltage was 10 kV, and the working distance was set to 10 mm to achieve optimal image resolution. All materials were cut into thin sections, coated with gold in a sputtering device and examined.

### 3.9. In Vitro Release of Curcumin (Cu/Cu*)

Prior to in vitro release of curcumin materials were rinsed with 70% ethanol and air dryed. Materials were weighted (100 mg) and immersed in a 25 mL phosphate buffer solution (PBS, pH = 7.4) maintained at 37 °C. Every 30 min, an equal volume of sample solution (2 mL) was taken and replaced with the same volume of fresh PBS. The absorbance at 420 nm was measured using a Jenway 6405 UV-VIS spectrophotometer. The drug release concentrations were assessed using calibration curves. The tests were conducted in triplicate, and the standard deviation (SD) was calculated.

### 3.10. Anti-Inflammatory Activity

In accordance with the procedure reported by Divakar et al. [45], with slight modifications, the anti-inflammatory effect of the samples was determined. A 100 mg portion of the tested materials was put in 5 mL of phosphate-buffered saline (PBS, pH 6.4) along with 2 mL of 0.1% bovine albumin solution. This mixture was first incubated at 37 °C for 15 min, then subjected to 70 °C for 5 min. After cooling, the absorbance at 660 nm was measured using PBS as a blank. All experiments were performed in triplicate, and the anti-inflammatory capacity was expressed as the percentage of inhibition calculated using equation (Equation (3)):

(3) $$\%Inhibition=100\times \left(1-\frac{A_{s}}{A_{c}}\right)$$

where *A_s_* and *A_c_* represent the sample and control absorbance values, respectively.

### 3.11. Antioxidant Activity

ABTS radical cations (ABTS•+) were prepared following a procedure adapted from Xiao et al. [46]. Specifically, a 7 mM solution of 2,2′-azino-bis(3-ethylbenzothiazoline-6-sulfonic acid) was combined in equal volume with a 2.45 mM potassium persulfate solution, and the mixture was left in the dark for 12 h. The resulting radical solution was then diluted in buffer at pH 7.4. For each test, 100 mg of the sample material was placed in a tube, followed by the addition of 4 mL of the ABTS radical solution. After a standard reaction period of 30 min the absorbance at 734 nm was measured. All measurements were carried out in triplicate. The scavenging efficiency was calculated according to Equation (4):

(4) $$Scavenging\;efficiency\;(\%)=\frac{A_{control}-A_{sample}}{A_{control}}\times 100$$

In this equation, *A_control_* is the absorbance of the control, which contains only free radicals and solvents. *A_sample_* is the absorbance of the sample in the presence of test compounds.

### 3.12. In Vitro Biocompatibility (MTS Assay)

Gingival fibroblasts (HGF, CLS Cell Lines Service GmbH, Eppelheim, Germany) were seeded (5 × 10^4^ cells/mL) into tissue culture-treated 96-well plates. Cells were incubated with fresh complete medium (Control) or diluted samples’ extracts (0.1/0.5/1 mg/mL) for 24 h. Biocompatibility of samples was assessed with the MTS assay, using the CellTiter 96^®^ AQueous One Solution Cell Proliferation Assay (Promega, Madison, WI, USA), according to the manufacturer’s instructions. Samples (10 mg/mL) were extracted over 24 h, at 37 °C, in complete cell culture medium: MEMα medium with 10% fetal bovine serum and 1% Penicillin-Streptomycin-Amphotericin B mixture (all from Gibco, Thermo Fisher Scientific, Waltham, MA, USA). Cells were incubated with fresh complete medium (Control) for 24 h. MTS absorbance at 490 nm was recorded on a FLUOstar^®^ Omega microplate reader (BMG LABTECH, Ortenberg, Germany). Experiments were done in triplicate and treated cell viability was expressed as percentage of Control cells’ viability (means ± standard error of the mean).

### 3.13. Statistical Analysis

One-way analysis of variance (ANOVA) was performed to evaluate differences among materials. When a significant effect was detected (*p* < 0.05), post-hoc comparisons were conducted using the Bonferroni correction. This method is particularly useful for controlling the error rate in multiple testing scenarios, ensuring that the probability of Type I errors—false positives, where a difference is detected when none actually exists—remains low. By adjusting the significance threshold for each comparison, the Bonferroni correction provides a more stringent criterion for detecting true differences between group means.

## 4. Conclusions

The results of this study highlight the potential of biopolymeric delivery systems based on chitosan, dextran/carboxymethyl dextran, lignin, and curcumin for biomedical applications. The mechanical evaluation revealed that incorporating curcumin significantly enhanced the diametral tensile strength (DTS) of the formulations, with Chi-mDex-L-Cu exhibiting the highest value (2.40 MPa), representing a 1233% increase as compared to its non-curcumin counterpart and 1200% higher than the baseline Chi-L. This significant reinforcement is attributed to the synergistic effect of curcumin and carboxymethyl dextran, which introduced additional binding sites through hydrogen bonding and electrostatic interactions, leading to a highly crosslinked and mechanically robust network. The structural characterization confirmed that dextran and mDex interfacial cohesion of the materials, with mDex-containing systems demonstrating a more uniform and compact morphology, further supporting their mechanical stability.

Curcumin release profiles varied significantly among the formulations, with Chi-L-Cu exhibiting the fastest release, followed by Chi-Dex-L-Cu, while Chi-mDex-L-Cu displayed a prolonged and controlled release profile. The Weibull model analysis showed that Chi-mDex-L-Cu had the lowest release rate (*k* = 0.00292) and asymptotic release (A = 177.37 µg/g), which was 55.66% lower than Chi-L-Cu and 54.06% lower than Chi-Dex-L-Cu. These differences underscore the role of carboxymethyl dextran in regulating curcumin diffusion by increasing matrix density through ionic crosslinking with chitosan. This extended release is particularly beneficial for biomedical applications requiring sustained therapeutic effects, such as chronic inflammatory diseases.

The antioxidant and anti-inflammatory properties demonstrated a strong correlation with curcumin release kinetics. Chi-L-Cu and Chi-Dex-L-Cu exhibited the highest initial antioxidant activities (~80% and ~65% inhibition, respectively) due to their faster curcumin diffusion, whereas Chi-mDex-L-Cu, despite its lower initial inhibition (~60%), is expected to maintain prolonged antioxidant activity over time. Similarly, the anti-inflammatory results showed that Chi-Dex-L-Cu achieved the highest inhibition (~90%), while Chi-mDex-L-Cu exhibited substantial bioactivity (~70%), evidencing the importance of a controlled curcumin release system for sustained anti-inflammatory effects. These findings highlight that mDex-based formulations offer an optimal balance between mechanical robustness, extended curcumin availability, and prolonged bioactivity, making them strong candidates for biomedical applications where controlled drug release is required.

The in vitro biocompatibility assessment confirmed the safety of all tested materials, with cell viability exceeding 85% across all formulations and concentrations. Chi-mDex-L-Cu exhibited a slight decrease in viability (~10% reduction at 1 mg/mL), likely due to increased polymer–cell interactions at higher concentrations, but remained well within the biocompatibility threshold, proving its suitability for further biomedical applications. The overall findings suggest that mDex-containing materials represent a versatile platform for sustained curcumin delivery with prolonged antioxidant and anti-inflammatory effects, excellent mechanical stability, and high biocompatibility, making them promising candidates for applications in wound healing, osteoarthritis treatment, and other chronic inflammatory conditions. Future in vivo studies will validate the therapeutic efficacy and long-term safety of these materials.

## Figures and Tables

**Table 1:** The Weibull parameters for the release of curcumin from the obtained materials.

Material	*A*	*x_c_*	*d*	*k*	R^2^
Chi-L-Cu	400 ± 12.5	−0.01457 ± 0.38	0.7103 ± 0.1318	0.00456 ± 0.00345	0.9949
Chi-Dex-L-Cu	386.11 ± 3.19	−1.55 × 10^−30^ ± 0.704 × 10^−16^	0.4999 ± 0.1182	0.00145 ± 0.0036	0.9966
Chi-mDex-L-Cu	177.36 ± 2.08	−1.909 × 10^−45^ ± 0.47 × 10^−29^	0.3717 ± 0.2275	0.00292 ± 0.01527	0.9893
Chi-L-Cu*	349.89 ± 15.63	−2.409 × 10^−5^ ± 0.0381 × 10^−11^	0.6971 ± 0.0946	0.00291 ± 0.00232	0.99635
Chi-Dex-L-Cu*	263.81 ± 11.20	−3.73426 × 10^−37^ ± 4.42552 × 10^−23^	0.3898 ± 0.2142	1.4897 × 10^−6^ ± 1.2701 × 10^−4^	0.98873
Chi-mDex-L-Cu*	215.00 ± 2.73	−8.60756 × 10^−41^ ± 1.53609 × 10^−24^	0.4151 ± 0.1233	0.00121 ± 0.00434	0.99484

**Table 2:** Sample formulations.

Component	Chitosan (g)	Dextran (g)	Carboxymethyl Dextran (g)	Lignin (g)	Curcumin (g)	Curcumin* (g)
Chi-L	0.25	-	-	0.1	-	-
Chi-Dex-L	0.25	0.1	-	0.1	-	-
Chi-mDex-L	0.25	-	0.1	0.1	-	-
Chi-L-Cu	0.25	-	-	0.1	0.1	-
Chi-Dex-L-Cu	0.25	0.1	-	0.1	0.1	-
Chi-mDex-L-Cu	0.25	-	0.1	0.1	0.1	-
Chi-L-Cu*	0.25	-	-	0.1	-	0.1
Chi-Dex-L-Cu*	0.25	0.1	-	0.1	-	0.1
Chi-mDex-L-Cu*	0.25	-	0.1	0.1	-	0.1

## Data Availability

The data presented in this study are available on request from the corresponding author.

## Supplementary Materials

The following supporting information can be downloaded at: https://www.mdpi.com/article/10.3390/molecules30061276/s1, Table S1: Analysis of Variance (ANOVA) of diametral tensile strength for the tested materials; Table S2: Pharmacokinetic parameters of curcumin release from polymeric matrices; Table S3: Analysis of Variance (ANOVA) of drugs release from the tested materials; Table S4: Analysis of Variance (ANOVA) of anti-inflammatory capacity for the tested materials; Table S5: Analysis of Variance (ANOVA) of antioxidant capacity for tested materials.
