# Supplementary material for: A Biopolymeric Dextran-Chitosan Delivery System for Controlled Release of Antioxidant and Anti-Inflammatory Compounds: Lignin and Curcumin
Source: Molecules. 2025 Mar 12;30(6):1276. doi: 10.3390/molecules30061276 (PMC11946708; doi:10.3390/molecules30061276)
Supplement: Supplementary file 1 [file molecules-30-01276-s001.zip › molecules-3477670-supplementary.pdf]

Supplementary Materials

A biopolymeric dextran-chitosan delivery system for controlled release of antioxidant and anti-inflammatory compounds: lignin and curcumin

Paula Cucu[1], Violeta Melinte[2], Anca Roxana Petrovici[2], Narcis Anghel[2,*], Irina Apostol[2], Mihai Mares[1], Natalia Simionescu[2] and Iuliana Spiridon[2,*]

[1]"Ion Ionescu de la Brad" University of Life Sciences, Faculty of Veterinary Medicine, Iasi, Romania;
[2]"Petru Poni" Institute of Macromolecular Chemistry, Iasi, Romania

Table S1. Analysis of Variance (ANOVA) of diametral tensile strength for the tested materials.

Groups	***Count***	***Sum***	***Average***	***Variance***		
(A): Chi-L	3	0.54	0.18	1.16×10^{-33}		
(B): Chi-Dex-L	3	0.35999	0.119997	0.00135		
(C): Chi-mDex-L	3	0.54	0.18	0.007351		
(D): Chi-L-Cu	3	0.45	0.15	0.00135		
(E): Chi-Dex-L-Cu	3	2.52	0.84	0.00375		
(F): Chi-mDex-L-Cu	3	7.32	2.44	0.0024		
Source of Variation	***SS***	***df***	***MS***	***F***	***P-value***	***F crit***
Between Groups	12.63866	5	2.527732	936.1433	4.09×10^{-15}	3.1058
Within Groups	0.032402	12	0.0027			
**Total**	12.67106	17				

where: SS – Sum of Squares, df – degree of freedom, MS – Mean Squares, F – Fisher's parameter, P – probability, Fcrit – critical value of Fisher's parameter

**POST HOC TEST**			**ALPHA**	
**Groups**	**p-value (t-test)**	**Significant?**	**Test**	**Alpha**
(A, B)	0.047411	NO	ANOVA	0.05
(A, C)	0.327625	NO	Bonferroni	0.000333333
(A, D)	0.607183	NO		
(A, E)	7.47×10^{-5}	YES		
(A, F)	3.83×10^{-6}	YES		
(B, C)	0.327625	NO		
(B, D)	0.373865	NO		
(B, E)	6.31×10^{-5}	YES		
(B, F)	3.23×10^{-7}	YES		
(C, D)	0.607183	NO		
(C, E)	0.000409	YES		
(C, F)	2.42×10^{-6}	YES		
(D, E)	7.47×10^{-5}	YES		
(D, F)	3.4×10^{-7}	YES		
(E, F)	3.83×10^{-6}	YES		

Table S2. Pharmacokinetic parameters of curcumin release from polymeric matrices.

Material	**AUC,** μg×min/g material	C_{max}, μg/g material	T_{max}, min
Chi-L-Cu	43950.0	250	270
Chi-Dex-L-Cu	29425.0	165	270
Chi-mDex-L-Cu	20025.0	110	270

Table S3. Analysis of Variance (ANOVA) of drugs release from the tested materials.

Groups	*Count*	*Sum*	*Average*	*Variance*		
Chi-L-Cu	3	850.4454	283.4818	28.469907		
Chi-Dex-L-Cu	3	542.0538	180.6846	11.643108		
Chi-mDex-L-Cu	3	330.408	110.136	1.7523934		
Source of Variation	***SS***	***df***	***MS***	***F***	***P-value***	***F crit***
Between Groups	45593.1356	2	22796.56783	1633.5610	6.16×10^{-09}	5.14325
Within Groups	83.7308	6	13.95513675			
**Total**	45676.8664	8				

where: SS – Sum of Squares, df – degree of freedom, MS – Mean Squares, F – Fisher's parameter, P – probability, Fcrit – critical value of Fisher's parameter

**POST HOC TEST**			**ALPHA**	
**Groups**	**p-value (t-test)**	**Significant?**	**Test**	**Alpha**
Chi-L-Cu vs Chi-Dex-L-Cu	0.00011669	YES	ANOVA	0.05
Chi-L-Cu vs Chi-mDex-L-Cu	0.000178503	YES	Bonferroni	0.01666667
Chi-Dex-L-Cu vs Chi-mDex-L-Cu	0.000291976	YES		

Table S4. Analysis of Variance (ANOVA) of anti-inflammatory capacity for the tested materials.

Groups	*Count*	*Sum*	*Average*	*Variance*		
(A): Chi-L	3	35.73	11.91	0.960247		
(B): Chi-Dex-L	3	41.16	13.72	1.815856		
(C): Chi-mDex-L	3	64.98	21.66	2.161092		
(D): Chi-L-Cu	3	109.26	36.42	7.327699		
(E): Chi-Dex-L-Cu	3	264.031	88.01033	10.93476		
(F): Chi-mDex-L-Cu	3	155.19	51.73	10.13936		
Source of Variation	*SS*	*df*	*MS*	*F*	*P-value*	*F crit*
Between Groups	12677.38	5	2535.477	456.308	2.99×10^{-13}	3.10587
Within Groups	66.67804	12	5.556504			
**Total**	12744.06	17				

where: SS – Sum of Squares, df – degree of freedom, MS – Mean Squares, F – Fisher's parameter, P – probability, Fcrit – critical value of Fisher's parameter

**POST HOC TEST**			**ALPHA**	
**Groups**	**p-value (t-test)**	**Significant?**	**Test**	**Alpha**
(A, B)	0.133038948	NO	ANOVA	0.05
(A, C)	0.002318314	YES	Bonferroni	0.000333333
(A, D)	0.000123092	YES		
(A, E)	2.79971×10^{-6}	YES		
(A, F)	3.21657×10^{-5}	YES		
(B, C)	0.002318314	YES		
(B, D)	0.000201884	YES		
(B, E)	3.5401×10^{-6}	YES		
(B, F)	4.48217×10^{-5}	YES		
(C, D)	0.001151005	YES		
(C, E)	5.86055×10^{-6}	YES		
(C, F)	0.00011973	YES		
(D, E)	3.09144×10^{-5}	YES		
(D, F)	0.003160428	YES		
(E, F)	0.000164978	YES		

Table S5. Analysis of Variance (ANOVA) of antioxidant capacity for tested materials..

Groups	*Count*	*Sum*	*Average*	*Variance*		
(A): Chi-L	3	225.27	75.09	2.195724		
(B): Chi-Dex-L	3	218.491	72.83033	2.344054		
(C): Chi-mDex-L	3	204.9	68.3	1.214473		
(D): Chi-L-Cu	3	228.69	76.23	3.374803		
(E): Chi-Dex-L-Cu	3	173.221	57.74033	4.334801		
(F): Chi-mDex-L-Cu	3	180	60	1.815856		
Source of Variation	*SS*	*df*	*MS*	*F*	*P-value*	*F crit*
Between Groups	929.6521	5	185.9304	73.01071	1.46×10^{-8}	3.10587
Within Groups	30.55942	12	2.546619			
**Total**	960.2116	17				

where: SS – Sum of Squares, df – degree of freedom, MS – Mean Squares, F – Fisher's parameter, P – probability, Fcrit – critical value of Fisher's parameter

**POST HOC TEST**			**ALPHA**	
**Groups**	**p-value (t-test)**	**Significant?**	**Test**	**Alpha**
(A, B)	0.140099808	NO	ANOVA	0.05
(A, C)	0.003117263	YES	Bonferroni	0.000333333
(A, D)	0.449885759	NO		
(A, E)	0.000299218	YES		
(A, F)	0.000199053	YES		
(B, C)	0.014149314	NO		
(B, D)	0.069518772	NO		
(B, E)	0.00053798	YES		
(B, F)	0.00040283	YES		
(C, D)	0.003040614	YES		
(C, E)	0.001483251	YES		
(C, F)	0.001172931	YES		
(D, E)	0.000322701	YES		
(D, F)	0.000247912	YES		
(E, F)	0.189674083	NO		
